# Does continuity of care impact decision making in the next birth after a caesarean section (VBAC)? a randomised controlled trial

**DOI:** 10.1186/1471-2393-13-140

**Published:** 2013-07-02

**Authors:** Caroline SE Homer, Karyn Besley, Jennifer Bell, Deborah Davis, Jon Adams, Alison Porteous, Maralyn Foureur

**Affiliations:** 1 Centre for Midwifery, Child and Family Health, Faculty of Health, University of Technology, Level 7, 235-253 Jones St. Broadway, Sydney, NSW, Australia; 2 Faculty of Health, University of Canberra, Canberra, Australia; 3 Maternity Unit, Gosford Hospital, Gosford, NSW, Australia

**Keywords:** Vaginal birth, Obstetrics, Caesarean section, Midwifery care, Vaginal birth after caesarean

## Abstract

**Background:**

Caesarean section (CS) has short and long-term health effects for both the woman and her baby. One of the greatest contributors to the CS rate is elective repeat CS. Vaginal birth after caesarean (VBAC) is an option for many women; despite this the proportion of women attempting VBAC remains low. Potentially the relationship that women have with their healthcare professional may have a major influence on the uptake of VBAC. Models of service delivery, which enable an individual approach to care, may make a difference to the uptake of VBAC. Midwifery continuity of care could be an effective model to encourage and support women to choose VBAC.

**Methods/Design:**

A randomised, controlled trial will be undertaken. Eligible pregnant women, whose most recent previous birth was by lower-segment CS, will be randomly allocated 1:1 to an intervention group or control group. The intervention provides midwifery continuity of care to women through pregnancy, labour, birth and early postnatal care. The control group will receive standard hospital care from different midwives through pregnancy, labour, birth and early postnatal care. Both groups will receive an obstetric consultation during pregnancy and at any other time if required. Clinical care will follow the same guidelines in both groups.

**Discussion:**

This study will determine whether midwifery continuity of care influences the decision to attempt a VBAC and impacts on mode of birth, maternal experiences with care and the health of the neonate. Outcomes from this study might influence the way maternity care is provided to this group of women and thus impact on the CS rate. This information will provide high level evidence to policy makers, health service managers and practitioners who are working towards addressing the increased rate of CS.

**Trial registration:**

This trial is registered with the Australian New Zealand Clinical Trials Registry (ANZCTR): ACTRN12611001214921

## Background

The rate of caesarean section (CS) in Australia has increased over the past decade and is now well above many similar countries [1,2]. One of the greatest contributors to the overall CS rate is a woman having an elective repeat CS (ERCS) [1]. In one Australian state, 76% of those with a previous CS who give birth at term have another CS, and one third of all CS involves women who have had a previous CS [1]. A National Review of Maternity Services undertaken in Australia highlighted concerns around the high rates of caesarean section and recommended that the Australian government “initiate targeted research aimed at improving the quality and safety of maternity services in select key priority areas, such as evidence around interventions, particularly caesarean sections” (page 14) [2].

Concerns about a high CS rate centre around growing evidence of increasing maternal morbidity associated with such operations for women, such as increased blood loss, blood clots, abdominal organ injury, need for hysterectomy and longer hospital stays [3-6]. Repeat CS has also been associated with significantly higher maternal and neonatal morbidity and mortality compared with caesarean or vaginal births for women who do not have a prior CS [5,7-10]. Babies born by CS are at risk of respiratory distress syndrome [11], persistent pulmonary hypertension and admission to a neonatal intensive care unit [12,13]. Neonatal mortality rates have been reported to be 1.7 to 1.9 times higher amongst babies born by CS compared with those born vaginally [14]. Previous CS has also been associated with an increased risk of stillbirth [15].

As an alternative, women who have had a previous lower-segment CS, and an uncomplicated pregnancy, can be offered to attempt a vaginal birth after CS (VBAC) [16]. However, a cross-sectional study undertaken in one Australian state (New South Wales) showed that, despite this, the rate of VBAC declined significantly between 1998 and 2006 (from 31% to 19%, respectively) and the proportion of women who attempted a vaginal birth also declined (from 49% to 35%, respectively) [17]. In this study, more than half the women (59%) who attempted VBAC were successful [17].

The best available international evidence suggests that VBAC does not increase the risk of hysterectomy or maternal mortality [18]. Neonatal mortality rate, after adjustments are made for pre-existing fetal anomalies, has been reported to be 24 per 10,000 for women who planned a VBAC compared with 9 per 10,000 for those who planned a repeat CS [18]. Although this results in an increased risk, the absolute risk is small, and comparable to the background risk of neonatal mortality for women having their first baby [19]. In the USA, a large prospective observational study involving nearly 46,000 women showed that planned VBAC was associated with a 1% increased risk of a blood transfusion, a 1% increased risk of endometriosis, and a 0.7% risk of symptomatic uterine rupture. Rates of uterine rupture for women who undergo a planned VBAC have been reported to be in the range of 22–74 per 10,000, compared with a risk of 0.5–2 per10,000 births in women with non-scarred uteri [1,19]. Other evidence shows a lesser increase in risk of perinatal mortality for VBAC, with a risk difference of 1.5 per 10,000 births compared with repeat caesarean section [20]. Therefore offering women the opportunity to attempt VBAC, in an appropriately staffed and resourced maternity unit, is a safe and ethical innovation to test.

Whereas no studies have examined the effects of midwifery continuity of care, some research has examined what influences women to choose VBAC. One study from the USA showed that the major influences were the woman’s sense of control in the decision-making process and the clinician’s encouragement of VBAC [21]. Whereas these were not linked to a model of care, it is well established that midwifery continuity of care programs increase women’s sense of control over decision making, [22] hence the logical link in our hypothesis. Two randomised controlled trials evaluating different antenatal education interventions designed to increase VBAC rates have been published and neither intervention altered VBAC rates [23,24]. One of the studies showed that consistent information using a decision-aid booklet reduced decisional conflict [25]. We plan to build on this evidence by providing consistent antenatal information from a small group of midwives, together with continuity of care in labour and birth.

### The intervention: midwifery continuity of care

Midwifery continuity of care allows women to develop a relationship with the same caregivers throughout pregnancy, birth, and the postnatal period. Women have a midwife caring for them during labour and birth whom they have met before and feel that they know, and this trusting relationship increases their confidence [26,27]. Women with a previous CS are often included in trials of midwifery continuity of care although they have not been specifically studied or targeted.

Midwifery continuity of care per se has been widely studied. A systematic review in the Cochrane Library examining midwifery continuity of care included 11 trials (12,276 women). Women who had midwife-led models of care were less likely to use regional analgesia (RR 0.81, 95% CI 0.73–0.91) or have an instrumental birth (RR 0.86, 95% CI 0.78–0.96), and were more likely not to use intrapartum analgesia/anaesthesia (RR 1.16, 95% CI 1.05–1.29), experience spontaneous vaginal birth (RR 1.04, 95% CI 1.02–1.06), feel in control during labour and childbirth (RR 1.74, 95% CI 1.32–2.30), and initiate breastfeeding (RR 1.35, 95% CI 1.03–1.76) [22]. Three trials have shown that midwifery continuity of care significantly reduced the CS rate: one small trial in Canada [28] and two in Australia, the most recent being published in 2012 [29,30].

In preliminary work for this trial, we reviewed the outcomes of a midwifery continuity of care program at one NSW hospital and compared the VBAC rate in the program with the rates in the area health service (AHS) in which the hospital is situated. The rate of attempted VBAC was 62.5% in the midwifery continuity of care program compared with 27% for the AHS and the vaginal birth rate was considerably higher in the program (76% and 54%, respectively). There was no increase in adverse outcomes for mothers or babies, although the total numbers in this evaluation were small (n = 377) (unpublished data). These data, although from a small number of women, provide some evidence to support the hypothesis to be tested in this trial, that is, that midwifery continuity of care will increase the proportion of women who attempt VBAC, increasing the overall rate of vaginal birth and reducing the CS rate.

## Methods/Design

The study uses a two arm, un-blinded randomised controlled design, to compare the outcomes for women who had midwifery continuity of care compared with those who had standard maternity care.

### Aim and objectives

The primary aim is to determine whether midwifery continuity of care for women with a previous CS increases the proportion of women who attempt vaginal birth in their current pregnancy. The primary hypothesis is that women with a previous CS, who are eligible for a vaginal birth and receive midwifery continuity of care, will be 50% more likely to choose to attempt a vaginal birth in their current pregnancy than similar women receiving standard care.

The secondary aims are to determine whether midwifery continuity of care increases the proportion of women experiencing a vaginal birth, and affects neonatal health or the emotional outcomes for women; and to explore the differences in women’s experiences of care and the decision making processes between the groups.

### Study population

All eligible women booking maternity care at two study sites in Australia will be invited to participate.

### Sample size

Our study of VBAC in NSW [17] showed that only 35% of women who are eligible for a vaginal birth following one previous CS attempted VBAC. The trial is designed to detect a clinically significant increase in the primary outcome, attempted VBAC, from 35% to 52.5%. In order to detect this difference 274 women will be required, providing 80% power with α = 0.05. To allow for a 15% loss to follow-up, a total of 332 women will be required, 166 in each group. This sample size will also be sufficient to demonstrate a significant improvement in VBAC success rates from 53% to 70.5% (n = 262).

The data informing the sample size calculations are drawn from a published cross-sectional study using population-based data from NSW [17,31] and descriptive data from the Obstetrix perinatal database at one of our study sites. Currently, midwives at this hospital providing continuity of care to women eligible for a VBAC are achieving attempt rates of 62.5% and success rates of 76%.

### Control and intervention groups

#### Intervention

##### Midwifery continuity of care (CofC)

Women allocated to the intervention group will receive midwifery continuity of care from a small group of midwives.[27] The midwives provide care during the antenatal, labour and birth, and postnatal periods (to two weeks postnatal). Midwives will adhere to The National Midwifery Guidelines for Consultation & Referral [32]. All women will have an appointment with an obstetric consultant during pregnancy (to re-assess VBAC suitability and discuss the birth plan). If a woman develops complications during pregnancy and requires additional care, she will continue with the midwifery continuity of care model while also attending obstetric or other consultations (this is the same for both groups). Labour and birth care will be provided at the hospital’s birth unit/labour ward and postnatal care will be provided in hospital or the woman’s own home following discharge from hospital.

#### Control

##### Standard midwifery care

Women allocated to the control group will receive the current model of public maternity care at the two study sites. Antenatal care is provided by antenatal staff (midwives and obstetricians). Staff in the Birth Unit provides labour and birth care and midwives in the postnatal ward provide postnatal care. Women are also offered midwifery visits at home following discharge from hospital (4–48 hours after a vaginal birth). All these care providers are different people.

### Inclusion criteria

● Most recent birth was by lower-segment CS

● No more than one previous CS

● Considered low risk, other than a history of one previous CS

● No other previous uterine incision

● No previous uterine rupture

● No contraindications for vaginal birth at the time of enrolment

● English proficiency (spoken and written)

● Public patient

● No known preference for a certain model of care, such as: GP-shared care or midwifery continuity of care

### Exclusion criteria

● Women who reside outside the hospital postnatal home-visiting zone

● Women who specifically request an ERCS at booking in

● Women with BMI > 35

### Trial recruitment

Women telephone the booking office to book for maternity care and receive an appointment for their first/booking-in visit. The women will be initially screened by the booking clerk for eligibility into the trial according to the eligibility criteria. An information pack about the study is posted to all eligible women. At the first/booking-in visit, the research assistant (RA) approaches each eligible woman and asks if she received the information and whether she is interested in participating. If the woman agrees, the consent form is signed and the woman is registered as a trial participant and the remote telephone allocation service at the university is contacted for random allocation.

### Group allocation

Randomisation will be on a 1:1 basis. Allocation concealment will be assured by using a remote telephone allocation service through the university research department. The RA will telephone the university to provide the woman’s initials, medical record number and date of birth. The clerk at the university campus will allocate women based on a randomization schedule developed independently from the RA. The midwife will be informed of the group to which the woman has been allocated and will receive her study number, which will be recorded in the Trial Register and Log Book. If allocated to standard care, the woman will be advised of her next clinic appointment. If allocated to midwifery continuity of care, she will be advised that the continuity of care midwives will contact her with a suitable appointment time.

### Data collection

#### Clinical data

The majority of clinical data required for the study are routinely collected and available in hospital records. The RA will collect these data and 5% of the records will be double checked by one of the CIs to verify their accuracy and consistency.

### Maternal

#### Demographic

● Age

● Parity

● Previous pregnancy outcomes

● Socio-economic status (derived from postcode, marital and employment status,)

● Past medical, surgical and obstetric risk factors

#### Pregnancy

● Complications during pregnancy

● Planned mode of birth prior to labour/CS

#### Labour and birth

● Syntocinon augmentation

● Artificial rupture of membranes

● Immersion in water for pain relief

● Epidural or spinal anaesthesia

● General anaesthetic

● Mode of birth

● Uterine rupture or scar dehiscence

● Major postpartum haemorrhage > 1000 mL and/or requiring operative procedure and/or blood transfusion

● Length of hospital stay

● Readmission Admission to ICU/HDU

● Maternal death (within 42 days)

#### Neonatal

● Apgar score < 7 at 5 min

● Admissions to neonatal unit within 48 hours of birth for at least 48 hours with feeding difficulties or respiratory distress

● Readmission to hospital

● Breastfeeding within 1 hour of birth

● Skin to skin contact within one hour of birth

● Mode of feeding at 6 weeks

● Stillbirth and neonatal death (within first 28 days)

#### Women’s experiences

Two questionnaires have been developed for administration at 36 weeks gestation (during pregnancy) and at 6–8 weeks post-partum (after the birth). These will enable women to report on their experiences with the model of care, their planned mode of birth (VBAC or CS) and the factors influencing the mode of birth choice. Distress and anxiety will be assessed using the Depression Anxiety Stress Scale (DASS 21) [33] and Edinburgh Postnatal Depression Scale (EDPS) [34]. Decisional conflict, knowledge, and anxiety will be assessed using measures from a previous Australian study [24].

The postnatal questionnaire includes questions about their labour and birth experiences and satisfaction with their decisions. With permission, we have adapted questions about the experience of care from surveys used to assess satisfaction in the COSMOS Study which examined the impact of caseload midwifery care on low risk women in Melbourne, Australia [29].

Both questionnaires were piloted with pregnant and postpartum women. Completion of each took approximately 20 minutes and they were reported to be understandable to the average adult reader.

#### Administration of questionnaires

The questionnaires have been professionally designed and printed. The women will be posted the questionnaires with a self-addressed envelope with a reply-paid stamp. The RA will contact participants to inform them that the questionnaire has been posted. The questionnaires will be linked to the participant by attaching the study code (documented in the log book) to the inside of the front cover of each booklet.

### Analysis

The analysis will be by ‘intention to treat’, including withdrawals and losses to follow-up. Randomisation should ensure that the groups are similar or equivalent in their baseline characteristics; additional multivariate analysis will be used if baseline differences are noted between the two groups. The relative risks (with 95% confidence intervals) of the primary outcomes will be calculated. Secondary outcome measures of categorical data will be analysed with χ^2^ tests and continuous data will be analysed with t-tests (for normally distributed data). Ranked or Likert-scale data will be analysed using cumulative odds ratios. Logistic regression and multiple linear regressions will be used if necessary to adjust for any other confounding variables.

It is not possible to blind participants to the model of care they receive, but outcome assessments will be blinded.

#### Interim analysis

A multidisciplinary data-monitoring group has appointed at the outset of the study to monitor the safety of the trial particularly examining differences between the groups that may be larger than expected and assessing any serious adverse effects that may occur. After 50% of the women have enrolled, a difference of at least three standard deviations in the interim analysis of a major endpoint will be needed to justify stopping the trial.

### Confidentiality and data security

All paperwork, documentation, internet and audiotaped data will be treated with confidentiality. The log books required on–site are kept in the office of the booking clerk. This office is staffed during business hours, and is only used by one staff member. The door is locked when the room is empty. Hard copies of client details and study matters are kept in a filing cabinet within the locked area at the university.

### Ethical aspects

Human Research Ethics Committee (HREC) approval for all current sites has been provided by the North Sydney Central Coast HREC, according to the single site HREC approval for multicentre clinical trials guidelines. Research governance approval has been provided by the Research Governance Office at each site.

### Data safety and monitoring

A Data and Adverse Event Monitoring Committee will assess the safety and serious adverse events and will be blinded to the assigned group. Serious adverse events will be given a Severity Assessment Code consistent with incident monitoring in NSW [35] and reviewed by the Committee. Any perinatal deaths will be analysed to determine whether the model of care contributed.

### Interruption of the study

The CIs may terminate this study prematurely, either in its entirety or either of the sites, for reasonable causes (e.g. unsatisfactory enrolment with respect to quantity or quality, inaccurate or incomplete data collection, falsification of records, failure to adhere to protocol). If this occurs, the site investigator will provide written notice to the CIs of the intended termination.

## Discussion

One of the greatest contributors to the overall CS rate is women having elective repeat CS. In this study, we will test the effects of midwifery continuity of care (where women have the same midwives through pregnancy and during labour and birth) on rates of vaginal birth and other outcomes in women with a prior CS. This will be the first Australian and international randomised controlled trial of midwifery continuity of care that focuses on women who have had a previous CS. High level evidence on midwifery continuity of care shows that these models increase women’s sense of control over decision making [22] hence the link in our hypothesis – that is, increased control will impact on decision making and increase rates of attempted vaginal birth.

Pregnancy, birthing and early parenthood are profoundly important life experiences that directly affect almost 300,000 families in Australia each year. An increasing CS rate is of national and international concern. The Editor of the prestigious journal Birth wrote in 2007 “reducing the caesarean birth rate worldwide is a complex and difficult task that must be tackled on many fronts using multiple strategies” [36]. A key finding of the Maternity Services Review in Australia was that the CS rate was higher than many similar countries and this highlighted the need for more research in this area [2]. The Australian National Maternity Services Plan [37] has also drawn attention to the fact that the high rates of caesarean section are often compounded by a lack of support for vaginal births after caesarean section providing preliminary evidence that midwifery continuity of care will indeed increase VBAC.

This will be the first national and international trial that has tested midwifery continuity of care as an intervention to increase vaginal birth rates in women who have had a previous CS. Although a number of studies have focused on interventions that improve the rates of attempted and/or successful VBAC, they have not been able to demonstrate their effectiveness. This study will provide this evidence that can be used to improve maternal outcomes while maintaining the safety of Australian mothers and babies.

## Competing interests

The authors declare they have no competing interests.

## Authors’ contributions

CH, MF and DD hypothesized the link between midwifery continuity of care and VBAC uptake and designed the randomised controlled trial and contributed to drafts of the paper. KB and JB project manage the trial and contributed to the drafts of the paper. JA provided input into the design of the qualitative aspects of the trial and contributed to drafts of the paper. AP provided input into the design of the trial and contributed to drafts of the paper. All authors read and approved the final manuscript.

## Study administration

### Principal investigators

Caroline Homer, Maralyn Foureur, Deborah Davis, Jon Adams, Alison Porteous.

### Associate investigators

Christine Roberts, Alison Shorten, Jennifer Fenwick.

### Steering committee members

Caroline Homer (Chair), Maralyn Foureur, Jenny Bell, Tracey Worrel, Lyndall Mollart, Kylie Normandale, Marian Bullard, Alison Porteous, Jane Knox, Bernadette Leiser, Mutayyab Shah, Angela Monger, Julie-Anne Olaisen.

## Pre-publication history

The pre-publication history for this paper can be accessed here:

http://www.biomedcentral.com/1471-2393/13/140/prepub
